# Late-onset anti-Yo antibody-positive paraneoplastic cerebellar degeneration: a case report

**DOI:** 10.3389/fsurg.2025.1676024

**Published:** 2026-01-13

**Authors:** Zhang Zhenyu, Guo Man, Zhao Guohui, Li Chenglong, Ma Yong, Zhou Jie, Cai Zhibiao

**Affiliations:** 1 Department of Neurosurgery, The 940th Hospital of Joint Logistics Support Force of the Chinese PLA, Lanzhou, Gansu, China; 2 First School of Clinical Medicine, Gansu University of Chinese Medicine, Lanzhou, Gansu, China

**Keywords:** anti-Yo antibody, ataxia, cancer, late-onset, ovarian, paraneoplastic cerebellar degeneration, vertigo

## Abstract

Anti-Yo antibody-positive paraneoplastic cerebellar degeneration (PCD) is a rare immune-mediated neurological syndrome associated with malignancy, presenting significant diagnostic and therapeutic challenges. This case describes an elderly female patient who developed delayed-onset subacute cerebellar symptoms three years after ovarian cancer resection, ultimately diagnosed with anti-Yo antibody-positive PCD. Although immunotherapy was administered, the patient's ataxia exhibited only limited improvement, suggesting that PCD may progress to an irreversible pathological stage. This case challenges the conventional understanding that PCD typically precedes tumour detection, offering a new perspective on clinical diagnosis and management due to the three-year interval. This case underscores the importance of considering paraneoplastic etiology in patients with unexplained neurological deficits who have a history of tumor surgery. Maintaining awareness for long-term screening for PCD and early management of primary malignancies alongside immunological interventions is crucial for delaying disease progression.

## Introduction

Paraneoplastic cerebellar degeneration (PCD) is a heterogeneous neurological syndrome associated with primary tumors. Currently, it is understood that the immune response against tumor antigens can cross-react with endogenous homologous antigens expressed in the nervous system, leading to immune-mediated damage to cerebellar Purkinje cells and often irreversible cerebellar dysfunction (1). Typical PCD usually presents with acute or subacute cerebellar symptoms such as ataxia, dysarthria, and nystagmus, and neurological impairment often precedes the diagnosis of the primary tumor. Diagnosis relies on the detection of highly specific anti-neuronal antibodies in serum and/or cerebrospinal fluid. Among them, anti-Yo antibody is an important biomarker for the diagnosis of PCD, the autoimmunity it mediates is highly aggressive, and its positivity is predominantly observed in patients with gynaecological malignancies or breast cancer (2). However, there exists a rare clinical scenario where neurological symptoms of PCD manifest several years after successful resection of the primary tumor. This article reports a case of a patient who developed delayed subacute cerebellar symptoms three years after radical surgery for ovarian cancer. After multiple referrals, this patient was ultimately diagnosed with anti-Yo antibody-positive PCD.

## Case report

**Figure 1:**
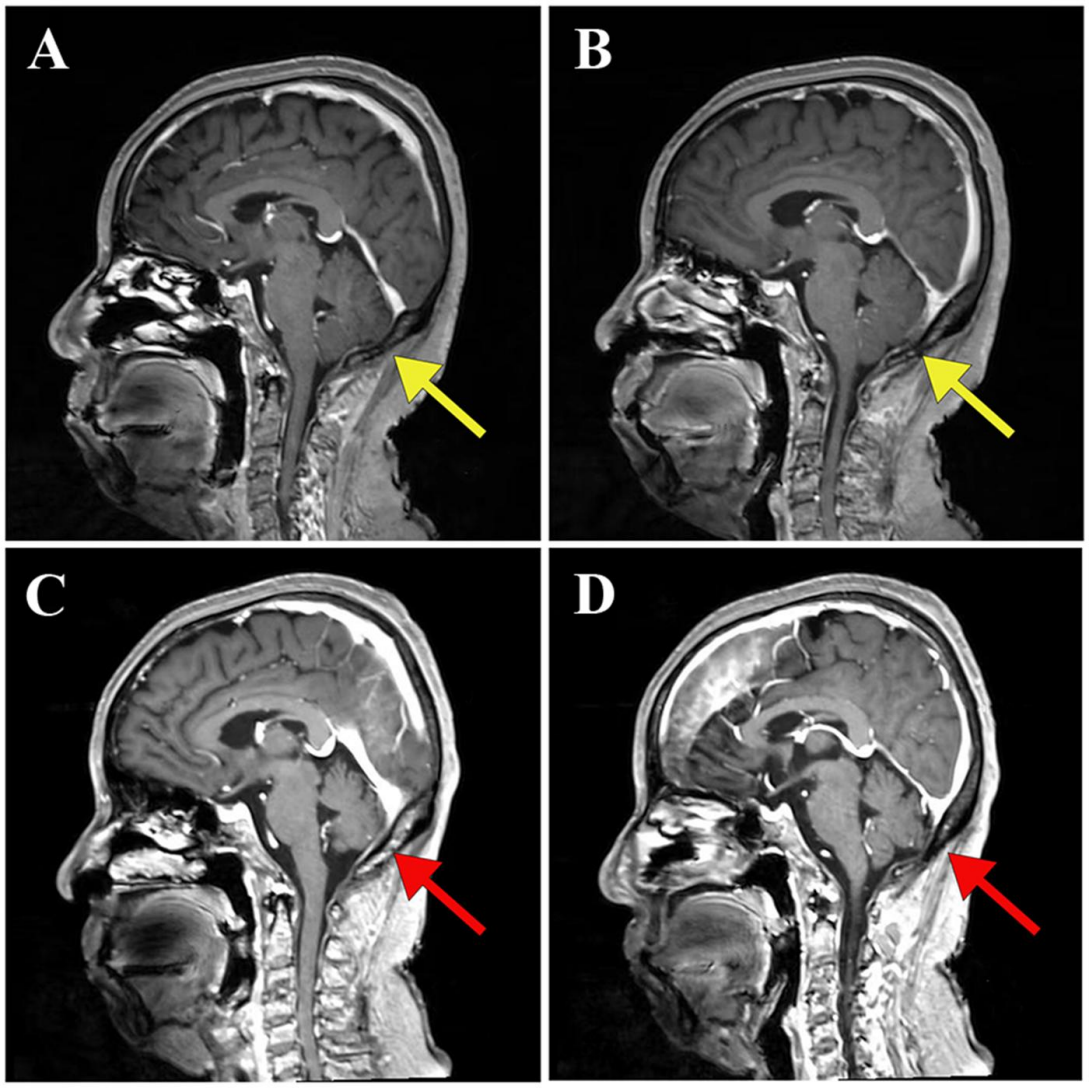
Cranial magnetic resonance imaging of a patient with PCD. **(A,B)** A cranial MRI conducted on 29 October 2024, revealed no significant abnormalities, as indicated by the yellow arrows. **(C,D)** A follow-up cranial MRI performed on 25 March 2025, demonstrated notable cerebellar atrophy, as highlighted by the red arrows.

A 70-year-old female patient presented with intermittent diplopia of insidious onset six months prior to admission (October 2024), accompanied by limb weakness and transient visual obscuration. She denied associated symptoms including headache, nausea, vomiting, limb convulsions, facial asymmetry, salivation, dysphagia, water brash, loss of consciousness, or incontinence. She was initially evaluated at an outside hospital and diagnosed with “ataxia syndrome”. Cranial magnetic resonance imaging (MRI) revealed no significant abnormalities (Figures 1A,B). Electronystagmography (ENG) demonstrated reduced low-frequency responses in the left horizontal semicircular canal. Symptomatic relief from medical management proved suboptimal. Following discharge from the initial facility, her condition progressed to include intermittent tremors of the head and bilateral upper extremities; these tremors were exacerbated by emotional arousal and intentional movements but were absent during sleep and accompanied by dizziness. She sought re-evaluation at another outside hospital where she was diagnosed with “metabolic encephalopathy.” Cerebrospinal fluid (CSF) analysis showed a white blood cell count of 10 × 10^6^/L and a protein level of 0.67 g/L. Autoantibody screening revealed a positive antinuclear antibody (ANA), positive ANA nuclear pattern, strongly positive Ro-52 (+++), and weakly positive ds-DNA (+/−). Tumor markers indicated a carcinoembryonic antigen (CEA) level of 1.56 ng/mL, which is below the normal threshold of <3.4 ng/mL. Subsequently, the patient's condition continued to decline, ultimately resulting in a bedridden state characterized by slurred speech, dysarthria, bradykinesia, and compromised fine motor skills. This deterioration necessitated her referral to our hospital for comprehensive evaluation.

### Past medical history

The patient was diagnosed with ovarian cancer in May 2021, for which she underwent radical debulking surgery and adjuvant chemotherapy. Histopathological examination confirmed high-grade serous carcinoma.

### Physical Examination

The patient was alert but exhibited a poor mental state, characterized by slurred speech and dysarthria. Her responses were slow; however, her answers were appropriate. Bilateral pupils were equal in size and round, exhibiting brisk light reflexes with a diameter of 3 mm. The convergence reflex was uncooperative. Ocular posture and eye movements appeared unremarkable. Bilateral hearing was approximately normal, and the crude olfactory function remained intact. The bilateral nasolabial folds were symmetric, with no deviation noted at the oral commissure. The tongue protruded midline, and forehead wrinkles displayed symmetry. The neck was soft without resistance observed upon palpation. Intermittent tremors were noted in the head as well as both upper limbs. Muscle tone was decreased overall, while muscle strength across all extremities measured at grade III. Pathological reflexes tested positive in the left lower limb, whereas tendon reflexes across all extremities were diminished; additionally, the pharyngeal reflex presented as weak.

### Auxiliary examination

**Figure 2:**
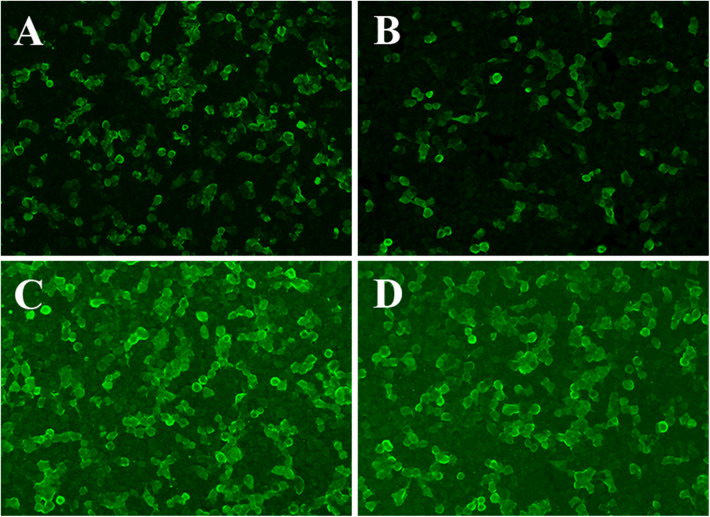
Patients with PCD tested positive for autoantibodies. **(A,B)** Positive detection of anti-Yo (CDR2) IgG and anti-Yo (CDR2L) IgG antibody in cerebrospinal fluid; **(C,D)** Blood tests revealed positive results for anti-Yo (CDR2) IgG and anti-Yo (CDR2L) IgG antibodies.

Cranial MRI (Figures 1C,D on 25 March 2025) revealed significant atrophy of the cerebellar cortex when compared to previous imaging studies. Blood and cerebrospinal fluid antibody tests returned positive results for anti-Yo antibodies (Figure 2).

### Diagnosis and treatment process

**Figure 3:**
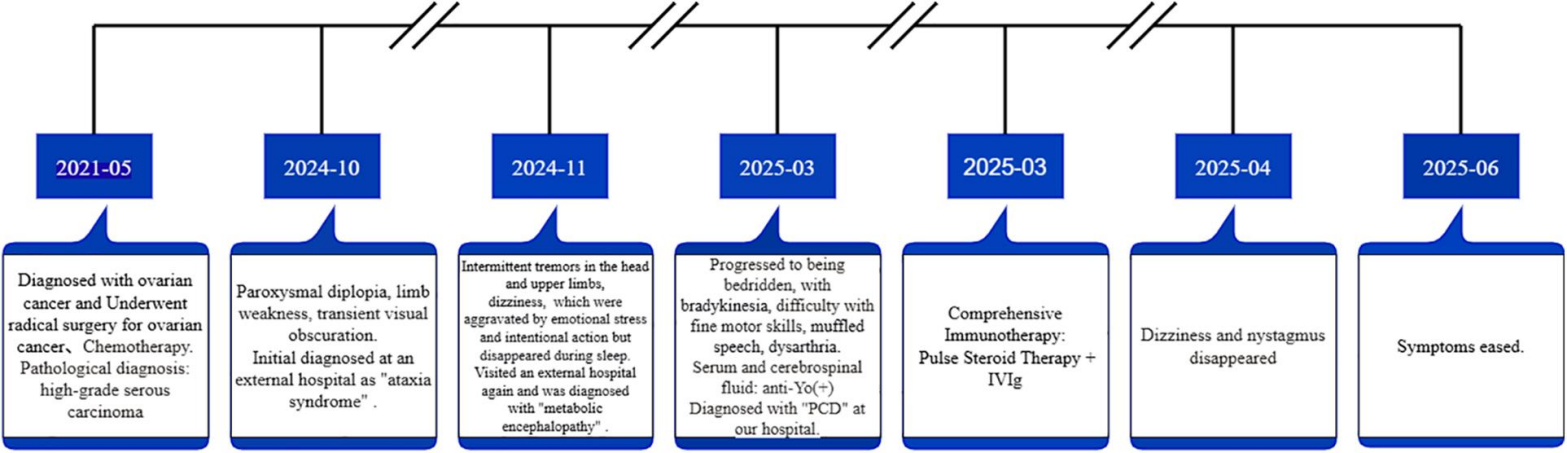
The completed timeline of diagnosis and treatment of this patient.

Based on the patient's history of ovarian cancer and auxiliary examinations, the diagnosis was PCD with positive anti-Yo antibody (Figure 3). In response to this condition, treatment commenced with glucocorticoid pulse therapy, utilizing methylprednisolone at a dosage of 1,000 mg/day for continuous intravenous infusion over three days, followed by a reduction to 500 mg/day for an additional three days. Concurrently, intravenous immunoglobulin (IVIg) was administered; based on the patient's weight of 62.5 kg, a total dose of 2 g/kg was given, amounting to 125 g in total, which was divided into five days of intravenous infusion at a rate of 25 g per day. Following the pulse therapy regimen, oral methylprednisolone tablets were prescribed starting at a dosage of 48 mg/day, with plans for tapering by one tablet every two weeks. Additionally, calcium carbonate D3 granules were taken orally twice daily at a dosage of 3 g to supplement calcium intake. Nifedipine controlled-release tablets were also continuously administered to manage and monitor blood pressure. One-month post-treatment initiation, the patient reported that dizziness had largely resolved; spontaneous nystagmus tests returned negative results and no nystagmus was observed during positional testing. Three months later during follow-up assessments, symptoms showed significant improvement with no reports of dizziness; however, some degree of ataxia persisted along with unclear but comprehensible speech. Continuous rehabilitation treatment remains ongoing.

## Discussion

Paraneoplastic Neurological Syndrome (PNS) is a well-defined “distant effect of cancer” that is extremely rare, It manifests as an autoimmune disease affecting both the central nervous system and the peripheral neuromuscular system, independent of tumor stage. The condition arises from the release of autoimmune antigens by tumor cells (1, 3). Among PNS manifestations, paraneoplastic cerebellar degeneration (PCD) characterized by positive anti-Yo antibodies is the most prevalent, particularly in patients diagnosed with breast and ovarian cancers. This syndrome correlates strongly with the presence of anti-Yo antibodies. Clinically, PCD often presents prior to tumor diagnosis and typically manifests as acute or subacute progressive cerebellar dysfunction. Initial symptoms frequently include nausea and vertigo, which subsequently progress to ataxia affecting both trunk and limbs, dysarthria, nystagmus, diplopia, and dysphagia (1, 4, 5). The clinical manifestations are diverse, these symptoms can develop rapidly within a timeframe ranging from weeks to months. In the absence of intervention, symptom severity tends to peak within six months. Patients frequently experience cognitive impairment, particularly memory loss, but subsequent dysarthria complicates accurate diagnosis of cognitive impairment; this progression may ultimately lead to disability (6). The pathogenesis of PCD has not been fully elucidated. It is generally believed to be caused by autoimmune reactions triggered by certain tumor components or by endocrine, metabolic, or nutritional disorders. Additionally, certain tumor-derived products exhibiting neurotoxicity have the potential to induce corresponding neuromuscular degeneration.

The diagnosis of PCD should always prioritize the search for primary malignant tumors, as tumors causing PCD can be very small, often presenting merely as regional lymphadenopathy (7, 8). Therefore, definitive tumor diagnosis necessitates imaging studies; early-stage head MRI may show no abnormalities. As the disease progresses, it may manifest as cerebellar atrophy, while PET-CT can reveal a reduced average metabolic rate in the cerebellum. However, there are case reports indicating that patients with anti-Yo antibody-positive PCD may exhibit widespread abnormal MRI signals in the cerebellar hemispheres during early stages (9).Initial cerebrospinal fluid analysis in PCD may suggest inflammation, and serum detection of corresponding antibodies can indicate paraneoplastic syndromes and guide the identification of potential tumors. PCD is an antibody-mediated autoimmune process associated with nearly 30 types of serological antibodies; among these, anti-Yo antibodies are the most common in PCD cases, accounting for approximately 50%, with a higher prevalence observed in females. Anti-Yo antibodies target two antigens: Cerebellar Degeneration-Related Protein 2 (CDR2) and CDR2-like protein (CDR2L). Both proteins belong to the family of cerebellar degeneration-related proteins and are primarily located within the cytoplasm and proximal dendrites of Purkinje cells. The immune system's attack on Purkinje cells leads to irreversible neuronal damage and subsequently results in progressive cerebellar ataxia (3, 10).

Currently, it has been observed that approximately 90% to 98% of patients with cerebellar ataxia who test positive for anti-Yo antibodies are found to have tumors, the majority of which are pelvic cancers and breast cancers, with occasional cases of lung cancer and Hodgkin's disease (11–13). In contrast, the occurrence of PCD in tumor patients with anti-Yo antibody positivity is exceedingly rare. A retrospective study by MONSTAD et al. (14) found that among 557 ovarian cancer patients and 253 breast cancer patients, the positivity rates for anti-Yo antibodies were 2.3% and 1.6%, respectively; furthermore, among those who tested positive for anti-Yo antibodies, 12% developed PCD. Therefore, under typical circumstances, the diagnosis of anti-Yo antibody-positive PCD precedes the diagnosis of the tumor, only a few cases present with delayed onset of anti-Yo antibody-positive PCD several years following tumor confirmation (15, 16). The uniqueness of this case lies in its three-year interval between onset and absence of tumor recurrence upon systematic evaluation. This indicates that even when primary tumors are well-controlled, there remains a possibility for the emergence of PCD. Additionally, neurological dysfunction caused by PCD may continue to progress independently from immune responses triggered solely by tumor recurrence. This provides a distinctive perspective on exploring the immunopathological mechanisms underlying PCD.

In this case, the patient exhibited typical symptoms of subacute cerebellar ataxia, with dizziness as the initial symptom. The patient sought medical attention multiple times at different hospitals but experienced a delay in diagnosis due to the absence of significant abnormalities on imaging studies and non-specific neurological findings. Upon admission to our hospital, comprehensive imaging examinations were conducted to rule out intracranial space-occupying lesions, meningeal and peripheral nervous system diseases, as well as infections. Considering the patient's history of ovarian cancer, targeted testing for anti-tumor syndrome-related antibodies was performed. High titers of anti-Yo antibodies were detected in both cerebrospinal fluid and serum, confirming the diagnosis of PCD. Additionally, cranial MRI showed cerebellar atrophy, consistent with the pathological changes of subacute cerebellar degeneration. Currently, there are no specific treatment methods for this condition; evidence-based treatment options for PCD remain limited. Consequently, therapeutic approaches largely rely on clinical experience. Treatment primarily focuses on immunotherapy (including corticosteroids, immunoglobulins, and immunosuppressants) while emphasizing early initiation of anti-tumor therapy to improve PCD symptoms; however, benefits to the nervous system are limited (13). Other studies have indicated that plasma exchange or administration of immunoglobulin or corticosteroids yield only mild to moderate efficacy, and intrathecal antibodies appear to be unaffected by plasma exchange or intravenous immunoglobulin. Most patients with positive anti-Yo antibodies have a poor overall prognosis, many progress to an inability to walk independently—less than 10% retain autonomous mobility (8). As most patients succumb due to complications related to neurological conditions, it is challenging to estimate the impact of anti-tumor treatments on survival outcomes. Early intervention is crucial for improving prognosis. Widdess-Walsh et al. (17) reviewed 15 cases of anti-Yo antibody-positive PCD treated with intravenous immunoglobulin (IVIG), within one month after symptom onset showed the best prognostic outcomes. Any delays may result in tumor progression and irreversible neurological damage leading to severe disability (18, 19). Therefore, enhancing subsequent rehabilitation efforts along with speech function restoration and psychological support can significantly aid patients in achieving optimal functional recovery.

## Conclusion

In summary, the patient in this case developed cerebellar ataxia symptoms three years after ovarian cancer resection, which is atypical compared to the characteristic early onset of paraneoplastic cerebellar degeneration (PCD) prior to tumor diagnosis. This clinical presentation is exceedingly rare. Future research should further explore the precise mechanisms by which the immune system continues to attack the nervous system in the absence of active tumors, potentially involving pathways such as persistent immune memory or prolonged exposure to previous tumor antigens. By analyzing the clinical features, diagnostic approach, and treatment response of this rare case, we emphasize that clinicians should maintain a high level of vigilance when encountering patients with a history of malignant tumors. Even if subacute cerebellar symptoms arise many years post-surgery without evidence of tumor recurrence, paraneoplastic etiologies must still be considered. Diagnosis requires a comprehensive assessment that includes detailed medical history taking, thorough physical examination, targeted antibody testing, and imaging evaluations to avoid misdiagnosis due to non-specific symptoms or extended time intervals. At the same time, it is essential to promote multidisciplinary collaboration and conduct regular assessments of tumor recurrence risk, monitor antibody titers, and evaluate cerebellar function. The aim is to enhance clinical awareness and attention towards patients exhibiting similar symptoms, enabling the timely initiation of treatment and rehabilitation to limit disease progression, improve quality of life, and provide valuable diagnostic and therapeutic insights for clinical practice.

## Data Availability

The raw data supporting the conclusions of this article will be made available by the authors, without undue reservation.
